# Long-Term Outcomes of First-Line Anti-TNF Therapy for Chronic Inflammatory Pouch Conditions: A Multi-Centre Multi-National Study

**DOI:** 10.3390/biomedicines13081870

**Published:** 2025-08-01

**Authors:** Itai Ghersin, Maya Fischman, Giacomo Calini, Eduard Koifman, Valerio Celentano, Jonathan P. Segal, Orestis Argyriou, Simon D. McLaughlin, Heather Johnson, Matteo Rottoli, Kapil Sahnan, Janindra Warusavitarne, Ailsa L. Hart

**Affiliations:** 1 Department of Gastroenterology, St Mark’s National Bowel Hospital & Academic Institute, London NW10 7NS, UK; ailsa.hart@nhs.net; 2 Department of Military Medicine, Faculty of Medicine, Hebrew University of Jerusalem, Jerusalem 9112102, Israel; fischmaya@gmail.com; 3 Surgery of the Alimentary Tract, IRCCS Azienda Ospedaliero-Universitaria di Bologna, 40138 Bologna, Italy; giacomo.calini2@unibo.it (G.C.); matteo.rottoli2@unibo.it (M.R.); 4 Department of Medical and Surgical Sciences, Alma Mater Studiorum University of Bologna, 40126 Bologna, Italy; 5 Department of Gastroenterology, Rambam Health Care Campus, Haifa 3109601, Israel; ed_koifman@rambam.health.gov.il; 6 Inflammatory Bowel Disease and Ileoanal Pouch Surgery Centre, Chelsea and Westminster Hospital, London SW10 9NH, UK; valerio.celentano@nhs.net; 7 Department of Surgery and Cancer, Imperial College London, London W12 0NN, UK; orestis.argyriou@nhs.net (O.A.); kapil.sahnan@nhs.net (K.S.); j.warusavitarne@nhs.net (J.W.); 8 Department of Gastroenterology, Royal Melbourne Hospital, Melbourne 3050, Australia; 9 Department of Medicine, University of Melbourne, Parkville 3010, Australia; 10 Department of Colorectal Surgery, St Mark’s National Bowel Hospital & Academic Institute, London NW10 7NS, UK; 11 Department of Gastroenterology, The Royal Bournemouth Hospital, Bournemouth BH7 7DW, UK; simon.mclaughlin@uhd.nhs.uk (S.D.M.); heather.johnson@uhd.nhs.uk (H.J.); 12 Department of Metabolism, Digestion and Reproduction, Imperial College London, London W12 0NN, UK

**Keywords:** adalimumab, infliximab, pouchitis, proctocolectomy, restorative, tumour necrosis factor inhibitors

## Abstract

**Background/Objectives:** Anti-tumour necrosis factor (anti-TNF) medications were historically commonly prescribed as the first-line biologic treatment for chronic inflammatory pouch conditions. However, their use in these conditions is mainly based on retrospective studies of relatively small numbers of patients with short follow up periods. We aimed to describe the long-term outcomes of first-line anti-TNF therapy in a large, multi-centre, multi-national patient cohort with chronic inflammatory pouch conditions. **Methods:** This was an observational, retrospective, multi-centre, multi-national study. We included patients with chronic inflammatory pouch conditions initially treated with anti-TNF drugs infliximab (IFX) or adalimumab (ADA), who had a follow up of at least 1 year. The primary outcome was anti-TNF treatment persistence, defined as continuation of anti-TNF throughout the study period. The secondary outcome was pouch failure, defined by the need for a defunctioning ileostomy or pouch excision. **Results:** We recruited 98 patients with chronic inflammatory pouch conditions initially treated with anti-TNF medications—63 (64.3%) treated with IFX and 35 (35.7%) treated with ADA. Average follow up length was 94.2 months (±54.5). At the end of the study period only 22/98 (22.4%) patients were still on anti-TNF treatment. In those in whom the first-line anti-TNF was discontinued, the median time to discontinuation was 12.2 months (range 5.1–26.9 months). The most common cause for anti-TNF discontinuation was lack of efficacy despite adequate serum drug levels and absence of anti-drug antibody formation (30 patients, 30.6%). Loss of response due to anti-drug antibody formation was the cause for discontinuation in 18 patients (18.4%), while 12 patients (12.2%) stopped treatment because of adverse events or safety concerns. Out of the 76 patients discontinuing anti-TNF treatment, 34 (34.7% of the cohort) developed pouch failure, and 42 (42.8% of the cohort) are currently treated with a different medical therapy. **Conclusions:** First-line anti-TNF therapy for chronic pouch inflammatory conditions is associated with low long-term persistence rates. This is due to a combination of lack of efficacy and adverse events. A significant percentage of patients initially treated with anti-TNF therapy develop pouch failure.

## 1. Introduction

Restorative proctocolectomy with ileal pouch–anal anastomosis (IPAA) is a common surgical option for patients with medically refractory ulcerative colitis (UC) [1]. However, this procedure is associated with several complications, notably pouchitis. Acute pouchitis affects approximately 60–80% of patients postoperatively [2,3], and of these, 10–15% may go on to develop chronic pouchitis [4,5].

In addition to chronic pouchitis, some patients develop Crohn’s disease (CD)-like features of chronic pouch inflammation, such as deep ulcerations, fistulisation, or pre-pouch ileitis (PPI) [6]. The aetiology and pathogenesis of these chronic inflammatory conditions remains largely unknown [7].

While most cases of chronic pouchitis initially respond to antibiotics [8,9,10], a subset of patients become antibiotic-dependent, requiring continuous or repeated treatment courses [11]. Furthermore, a smaller group develops antibiotic-refractory inflammation, often necessitating escalation to biologic therapies [12]. Among biologics, vedolizumab is the only agent that has been evaluated in a randomised controlled trial specifically for chronic pouchitis [13]. Despite this, anti-TNF agents such as infliximab (IFX) and adalimumab (ADA) are frequently used off-label for chronic inflammatory pouch conditions [10].

The evidence supporting anti-TNF use in this setting is largely derived from small, single-centre retrospective studies with limited follow-up durations [14,15,16,17]. These studies suggest that both IFX and ADA can achieve short-term clinical responses in a substantial proportion of patients with inflammatory disorders related to the ileal pouch [18]. However, larger and more robust data are needed to validate these findings and better inform clinical decision-making.

Our group previously reported outcomes from a single-centre cohort of 34 patients with chronic inflammatory pouch conditions treated with IFX as first-line biologic therapy. In that cohort, IFX achieved clinical effectiveness in about 50% of cases at one year, with most non-responders avoiding ileostomy through subsequent biologic therapies [19].

Despite these early observations, there remains a significant gap in long-term, multi-centre data regarding the outcomes of anti-TNF therapy for chronic inflammatory pouch conditions.

This study aimed to assess the long-term treatment persistence and pouch failure rates of first-line anti-TNF treatment in a large, multi-centre, multi-national cohort. To our knowledge, this represents the largest and most comprehensive study on this topic to date.

## 2. Materials and Methods

This was a retrospective, multi-centre, multi-national observational study. Data were collected from three centres in the United Kingdom, one centre in Haifa, Israel, and one centre in Bologna, Italy. The study included three tertiary referral centres and two district general hospitals.

Patients were censored at the last clinic visit following their most recent biologic therapy or the development of pouch failure.

**Inclusion and Exclusion Criteria**:

Inclusion criteria included the following:

- Undergone ileal pouch–anal anastomosis (IPAA) with formation of a J-pouch for ulcerative colitis (UC);
- Evidence of chronic inflammatory pouch condition on endoscopic assessment, with inflammation confirmed histologically;
- Received antibiotic therapy prior to starting anti-TNF treatment;
- Diagnosed with chronic antibiotic-refractory pouchitis (CARP);
- Treated with at least one dose of infliximab (IFX) or adalimumab (ADA) for post-colectomy chronic inflammatory pouch condition;
- No other post-colectomy biologic treatments before initiating anti-TNF therapy;
- Minimum follow-up of one year after anti-TNF treatment initiation;
- One of the following reported outcomes within the last two months of their final IFX infusion or the last two weeks of their final ADA injection:
  ○ Pouch failure, defined as the need for a defunctioning ileostomy;
  ○ Switch to another advanced inflammatory bowel disease (IBD) medication (either an in-class switch to another anti-TNF or a switch to a different drug class) due to lack of efficacy (either primary non-response or secondary loss of response);
  ○ Discontinuation of anti-TNF due to antibody formation, allergic reaction, treatment-related adverse events, or safety concerns;
  ○ Advised to continue anti-TNF therapy at the last infusion or injection.

Exclusion criteria included the following:

- Patients who underwent IPAA for familial adenomatous polyposis (FAP) or Crohn’s disease;
- Patients lost to follow-up.

Golimumab and certolizumab pegol were not included in the study, as no patients receiving these medications were identified in our hospitals’ pouch patient databases.

Anti-drug antibody levels and serum trough levels were not routinely measured across all participating centres, but were recorded when available.

Pouchitis was defined using the Pouch Disease Activity Index (PDAI) [20]. Patients were classified as having pouchitis if their PDAI score within one year before initiating anti-TNF therapy was ≥7.

Pre-pouch ileitis (PPI) was defined as inflammation immediately proximal to the pouch inlet, as evidenced by endoscopic findings such as oedema, erythema, ulceration, or contact bleeding.

The primary outcome was anti-TNF treatment persistence, defined as continuation of the same anti-TNF agent throughout the study period.

The secondary outcome was pouch failure, defined as the need for a defunctioning ileostomy, with or without pouch excision.

All investigators used a standardised Excel spreadsheet for data collection.

### Statistical Methods

Descriptive statistics were used to summarize baseline characteristics of the cohort, with continuous variables expressed as medians with interquartile ranges (IQRs), and categorical variables as counts and percentages. Time-to-event outcomes, including time to therapy discontinuation and time to pouch failure, were analysed using Kaplan–Meier survival curves. To evaluate the association between the anti-TNF agent type and these outcomes, hazard ratios (HRs) and corresponding 95% confidence intervals (CIs) were estimated using univariate Cox proportional hazards models. Statistical significance was defined as a two-tailed *p*-value less than 0.05.

All statistical analyses were performed using R, version 4.1.3 (R foundation for statistical computing, Vienna, Austria).

Ethical approval was granted by the Health Research Authority.

The study was carried out in accordance with the principles of the declaration of Helsinki.

## 3. Results

### 3.1. Patient Characteristics

We recruited 98 patients with chronic inflammatory pouch conditions initially treated with anti-TNF medications. The average age of UC diagnosis was 24.0 years (±10.5 years), and the average patient age at study inclusion was 51.3 years (±13.8 years). Total follow-up averaged 94.2 months (±54.5 months). Nine patients had anti-TNF treatment (IFX in all cases) prior to colectomy, and 12 (12.2%) patients were smokers (Table 1 and Supplementary Table S1).

The most common indication for anti-TNF treatment was chronic pouchitis with concomitant pre-pouch ileitis (66/98 patients, 67.3% of the cohort). Chronic pouchitis without pre-pouch ileitis was the treatment indication in 25 patients (25.5% of the cohort). Pre-pouch ileitis, without concomitant pouchitis, was seen in 8 patients (8.2% of the cohort). Cuffitis was present in 14 patients (14.3%), and 20 patients (20.4%) had pouch-related fistulae. Strictures in the pre-pouch ileum or pouch inlet were present in 45 patients (45.9%).

IFX was the most common first-line anti-TNF treatment (63/98 patients, 64.3%), while 35 patients (35.7%) were treated with first-line ADA. Anti-TNF was administered as monotherapy in 57 patients, while 27 patients received it in combination with an immunomodulator. In 14 patients, data on whether they received monotherapy or combination therapy was not available.

Second-line advanced medical treatments in our cohort included IFX (6 patients), ADA (15 patients), vedolizumab (16 patients), ustekinumab (13 patients), and upadacitinib (1 patient).

### 3.2. Anti-TNF Discontinuation

At the end of the study period only 22/98 patients (22.4%) were still on first-line anti-TNF treatment.

Endoscopic and histologic evaluation of the pouch post anti-TNF treatment was available for 19 of these patients. Since it is a retrospective study, these evaluations were not performed at an identical scheduled time, but rather at the discretion of the patients’ gastroenterologist. Five patients were in endoscopic and histologic remission, two were in endoscopic remission but with ongoing inflammation on histology, and twelve had active inflammation on endoscopy and histology.

**Figure 1:**
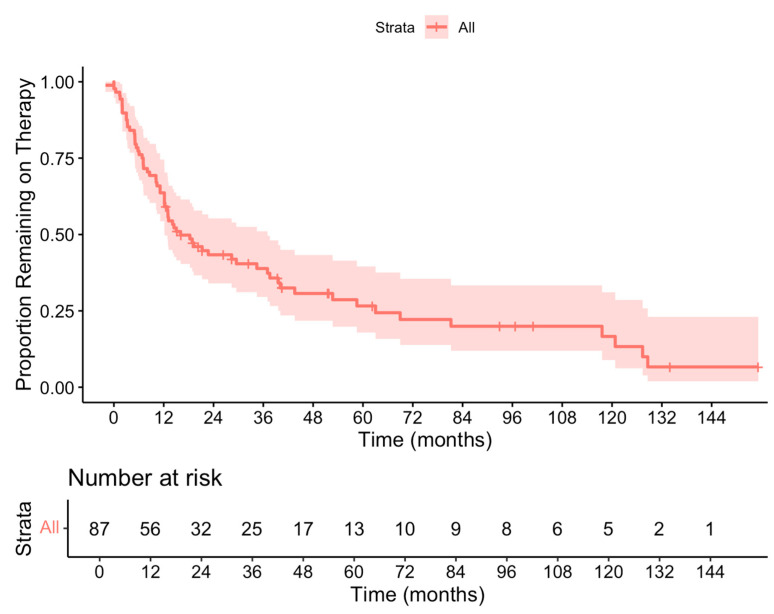
Kaplan–Meier plot showing the proportion of patients without anti-TNF discontinuation.

In those in whom the first-line anti-TNF was discontinued, the median time to discontinuation was 12.2 months (range 5.1–26.9 months). A graphical illustration of time to first-line anti-TNF discontinuation is shown in Figure 1.

The most common cause for anti-TNF discontinuation was lack of efficacy despite adequate serum drug levels and absence of anti-drug antibody formation (30 patients, 30.6%). It is worth noting that in 17/30 of these patients (56.7%) an attempt to optimize anti-TNF treatment was made prior to discontinuation. Loss of response due to anti-drug antibody formation was the cause for discontinuation in 18 patients (18.4%), while 12 patients (12.2%) stopped treatment because of adverse events or safety concerns. The most common adverse events leading to treatment discontinuation were rash and arthralgia. More significant side effects included immediate infusion reactions, peripheral neuropathy, recurrent pneumonia, and osteomyelitis. In 16 patients, data on the cause of anti-TNF treatment discontinuation was not available.

Compared to patients receiving adalimumab, patients with infliximab had an HR of 2.0 for treatment discontinuation (95% CI, 1.1–3.5, *p* = 0.019), see Table 2.

Compared to patients receiving combination therapy of anti-TNFs with immunomodulators, patients receiving anti-TNF monotherapy had similar rates of treatment discontinuation (HR = 1.3, 95% CI, 0.71–2.2, *p* = 0.43).

There were no statistically significant differences in treatment discontinuation rates between patients undergoing colectomy for medically refractory disease and patients undergoing colectomy for dysplasia (HR = 2.84, 95% CI, 0.39–23.13, *p* = 0.42).

There were no statistically significant differences in treatment discontinuation rates between patients treated at tertiary referral centres and patients treated at district general hospitals (HR = 1.56, 95% CI, 0.37–6,61, *p* = 0.9).

### 3.3. Second-Line Therapy Outcomes

Out of the 76 patients who discontinued first-line anti-TNF treatment, 42 (42.8% of the cohort) are currently treated with a different medical therapy, and 34 (34.7% of the cohort) developed pouch failure.

Second-line biologic persistence rates were 0/6 (0%) for second-line infliximab, 1/15 for second-line adalimumab (6.7%), 5/16 (31.2%) for second-line vedolizumab, 3/13 (23.1%) for second-line ustekinumab, and 1/1 (100%) for second-line upadacitinib.

### 3.4. Pouch Failure

**Figure 2:**
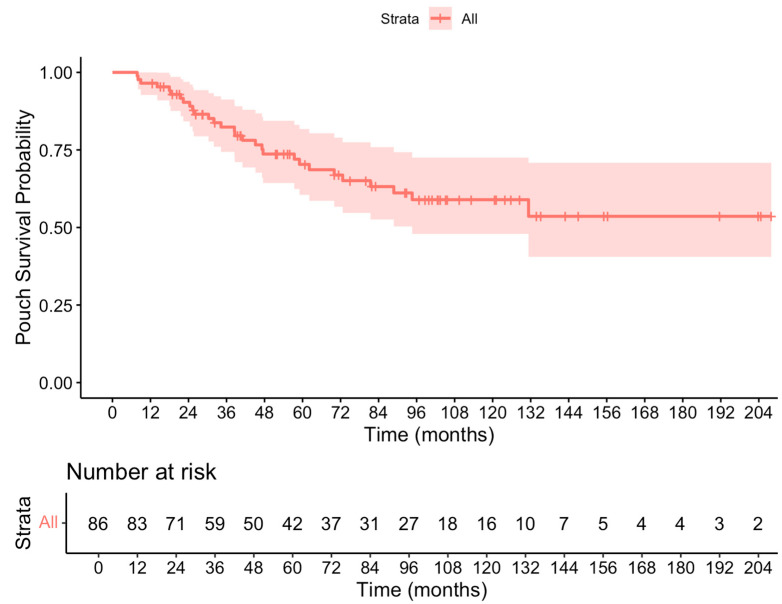
Kaplan–Meier plot showing 10-year ileostomy-free survival.

In those who developed pouch failure, the median time to pouch failure in those who developed it was 38.5 months (range 19.8–58.6 months). Figure 2 illustrates the time to pouch failure.

Patients receiving infliximab were not at increased risk of pouch failure compared to those receiving adalimumab (HR 1.9, 95% CI; 0.78–4.7, *p* = 0.16).

## 4. Discussion

To the best of our knowledge, this is the largest study with the longest follow-up data exploring the outcomes of first-line anti-TNF therapy for chronic inflammatory pouch conditions, with an average follow-up length of almost eight years [16,19,21,22].

We found that first-line anti-TNF therapy is associated with very low long-term persistence rates. The persistence rates in our study were lower than in previous studies on anti-TNF treatment [16,21,22]. This might be explained by the significantly longer follow up period in our cohort, as persistence rates of anti-TNF medications are known to decrease over time [23]. Another potential explanation is the relatively high proportion of patients with pre-pouch ileitis in our cohort. This condition has been previously shown to be associated with worse clinical outcomes, such as failure of biologic treatment to induce endoscopic remission [24] and high rates of pouch failure [25]. Furthermore, our study included patients treated at tertiary referral centres, which may result in a referral bias, and potentially account for these very low persistence rates.

An interesting finding in our study is the significantly higher treatment discontinuation rates among patients treated with IFX compared to ADA.

This observation is in contrast with a 2021 meta-analysis, which found IFX to be associated with higher rates of clinical improvement and clinical remission than ADA in patients with chronic antibiotic refractory pouchitis [26]. These differences might be explained by the relatively low rates of patients receiving IFX in combination with an immunomodulator in our cohort. While data specifically focusing on IFX in combination with immunomodulators in the context of chronic inflammatory pouch conditions are scarce, there is a solid evidence base supporting the superior efficacy of this combination compared to IFX monotherapy in both UC [27] and Crohn’s disease [28]. Future head-to-head randomised controlled trials, comparing IFX vs. ADA and anti-TNF monotherapy vs. combination therapy with immunomodulators in patients with chronic inflammatory pouch conditions, are sorely needed to help clarify these issues.

In addition to the low persistence rates of first-line anti-TNF therapy, a significant proportion of patients in our cohort (34.7%) who were initially treated with anti-TNFs went on to experience pouch failure. The reported incidence of pouch failure following an IPAA for UC treatment is 5% at 5 years and ranges from 8% to 15% after 10 to 20 years [29,30,31]. This indicates that the pouch failure rate in this patient group is notably higher compared to the overall pouch failure rates seen in UC patients undergoing an IPAA. These concerning figures suggest that different treatment approaches may be needed for this distinct and complex patient group.

From a clinical practice perspective, anti-TNFs carry significant safety concerns, including increased risks of infections and malignancies [32,33,34,35]. The high discontinuation rate of anti-TNF treatments in our cohort highlights the need for careful consideration of alternative treatment options and potential complications. Unfortunately, despite the continuous expansion of medical treatment options for inflammatory bowel diseases, vedolizumab remains the only medication to be formally studied in a randomised controlled trial in patients with chronic inflammatory pouch conditions [13]. We hope to see additional medications being studied specifically in this unique patient population.

Our findings suggest that anti-TNFs might not be an ideal first-line biologic treatment for chronic inflammatory pouch conditions. A biologic such as vedolizumab, which has proven efficacy in this patient group [13], may be preferable. Our study also raises the possibility that, in some patients starting anti-TNFs for chronic inflammatory pouch conditions, we may be merely postponing an inevitable pouch failure and ileostomy, rather than modifying disease course. This notion is strengthened by the low persistence rates for second-line biologic treatments in our cohort.

This study has several limitations. The majority of the patients were recruited from tertiary referral centres. Some of these centres also receive a high volume of referrals regarding management of patients with pouch-related complications. Thus, our findings reflect outcomes in patients with chronic inflammatory pouch conditions that are managed in high-volume specialist pouch centres. These might not be generalisable to centres that manage smaller numbers of patients with pouch-related complications.

Additionally, it is a retrospective study, and includes all the limitations associated with a retrospective historical cohort study design, such as the potential presence of confounding factors and biases and the inability to determine causation. Future studies should ideally assess anti-TNF treatment for chronic inflammatory pouch conditions in prospective randomised controlled trials.

Another limitation of our study is that in some patients (16/76 patients discontinuing treatment) we were unable to determine the cause of anti-TNF treatment discontinuation from their medical records, due to lack of information regarding anti-TNF serum drug levels and presence of anti-drug antibodies. However, a recent multi-centre study of patients with chronic pouchitis showed that this information might not be as valuable as some might believe, since higher anti-TNF serum concentrations were not associated with more clinical or endoscopic remission [36]. We also lacked information on whether anti-TNFs were used as monotherapy or in combination with an immunomodulator in 14/98 patients, which may have impacted the results of our subgroup analysis comparing treatment outcomes of monotherapy versus combination therapy.

Furthermore, while we were able to document the presence of pouch-related fistulae in our cohort, we lacked more granular data regarding fistulae type and anatomy, which can possibly affect clinical outcome.

Another aspect lacking in our study, and worth considering in future studies, is quality of life assessment, for example when comparing patients that continue on anti-TNF medications against those who opt for early ileostomy formation.

## 5. Conclusions

In conclusion, we have demonstrated that first-line anti-TNF therapy for chronic pouch inflammatory conditions is associated with low long-term persistence rates. This is due to a combination of lack of efficacy and adverse events. A significant percentage of patients initially treated with anti-TNF therapy develop pouch failure. These patients should be counselled about the high risk of pouch failure.

## Figures and Tables

**Table 1:** Baseline characteristics.

Characteristic	*n* = 98
Age (years)	51.3 (37.5–65.1)
Age at UC diagnosis (years)	24 (13.5–34.5)
Ethnicity	
Caucasian	77/98 (78.6%)
Middle Eastern	9/98 (9.2%)
South Asian	12/98 (12.2%)
Females	45/98 (45.9%)
Smoking	12/98 (12.2%)
PSC	4/98 (4.1%)
Colectomy indication	
Refractory disease	90/97 (92.8%)
Dysplasia	7/97 (7.2%)
Missing	1
Anti-TNF treatment prior to colectomy	
Yes	9/88 (10.2%)
No	79/88 (89.8%)
Missing	10
Small bowel strictures	45/98 (45.9%)
Cuffitis	14/98 (14.3%)
Fistulae	20/98 (20.4%)
Anti-TNF indication	
Isolated PPI	7/98 (7.1%)
PPI and pouchitis	66/98 (67.3%)
Isolated pouchitis	25/98 (25.6%)
First-line Anti-TNF	
ADA	35/98 (35.7%)
IFX	63/98 (64.3%)
Combo or monotherapy	
Combo	27/84 (32.1%)
Monotherapy	57/84 (67.9%)
Missing	14

**Table 2:** Outcomes of infliximab vs. adalimumab.

	Infliximab	Adalimumab	
*n*	63	35	
Discontinuation rate	50/63 (79.4%)	26/35 (74.3%)	***p* = 0.019**
Pouch failure rate	23/63 (36.5%)	10/35 (31.4%)	*p* = 0.16

## Data Availability

Dataset available on request from the authors.

## Supplementary Materials

The following supporting information can be downloaded at: https://www.mdpi.com/article/10.3390/biomedicines13081870/s1, Table S1: Raw data used in all tables and figures.
